# An assessment of yield gains under climate change due to genetic modification of pearl millet

**DOI:** 10.1016/j.scitotenv.2017.06.002

**Published:** 2017-12-01

**Authors:** Piara Singh, K.J. Boote, M.D.M. Kadiyala, S. Nedumaran, S.K. Gupta, K. Srinivas, M.C.S. Bantilan

**Affiliations:** a International Crops Research Institute for the Semi-Arid Tropics (ICRISAT), Patancheru 502 324, Andhra Pradesh, India; b Agronomy Department, University of Florida, IFAS, Gainesville, FL 32611-0500, USA

**Keywords:** Traits evaluation, Genetic improvement, Pearl millet model, Abiotic stresses, Climate change adaptation

## Abstract

Developing cultivars with traits that can enhance and sustain productivity under climate change will be an important climate smart adaptation option. The modified CSM-CERES-Pearl millet model was used to assess yield gains by modifying plant traits determining crop maturity duration, potential yield and tolerance to drought and heat in pearl millet cultivars grown at six locations in arid (Hisar, Jodhpur, Bikaner) and semi-arid (Jaipur, Aurangabad and Bijapur) tropical India and two locations in semi-arid tropical West Africa (Sadore in Niamey and Cinzana in Mali). In all the study locations the yields decreased when crop maturity duration was decreased by 10% both in current and future climate conditions; however, 10% increase in crop maturity significantly (p < 0.05) increased yields at Aurangabad and Bijapur, but not at other locations. Increasing yield potential traits by 10% increased yields under both the climate situations in India and West Africa. Drought tolerance imparted the lowest yield gain at Aurangabad (6%), the highest at Sadore (30%) and intermediate at the other locations under current climate. Under climate change the contribution of drought tolerance to the yield of cultivars either increased or decreased depending upon changes in rainfall of the locations. Yield benefits of heat tolerance substantially increased under climate change at most locations, having the greatest effects at Bikaner (17%) in India and Sadore (13%) in West Africa. Aurangabad and Bijapur locations had no yield advantage from heat tolerance due to their low temperature regimes. Thus drought and heat tolerance in pearl millet increased yields under climate change in both the arid and semi-arid tropical climates with greater benefit in relatively hotter environments. This study will assists the plant breeders in evaluating new promising plant traits of pearl millet for adapting to climate change at the selected locations and other similar environments.

## Highlights

• Climate change will adversely impact pearl millet yields in India and West Africa.
• Changing crop maturity duration did not benefit crop yields at the warmer sites.
• Drought and heat tolerance traits increased yields at the dry and warmer sites.
• Millet model was useful to assess benefits of genetic traits under climate change.

## Graphical abstract

**Figure:**
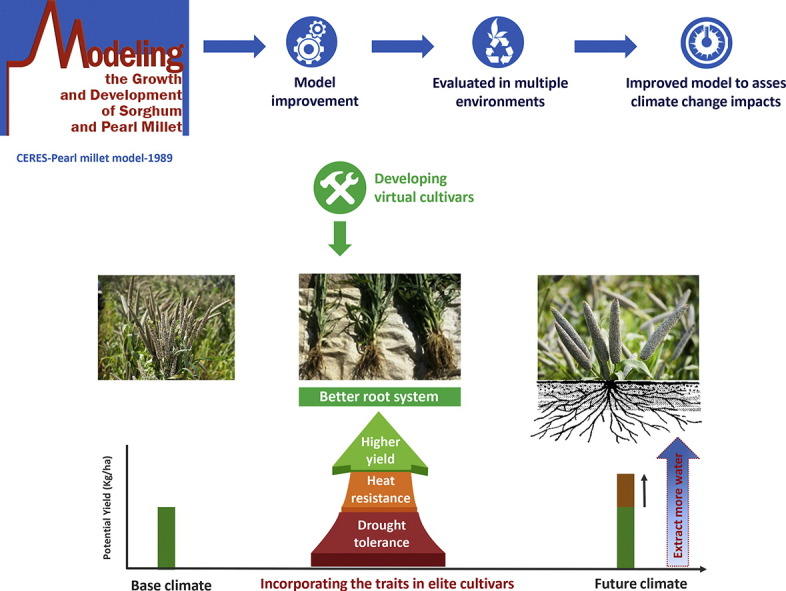

## 1 Introduction

Millets are an important source of food for humans and feed and fodder for animals in the arid and semi-arid tropical regions of Asia and Africa. Considering cultivated area and production, finger millet, pearl millet, foxtail millet and proso millet are the most important millets. Pearl millet accounts for > 50% of the global millet production. In Asia, it is mainly grown in South and East Asia and in Africa, mainly in the Sahel. Pearl millet occupies > 95% of the total millet area in Africa (16.8 million ha). The West and Central African region has the largest area under millets and the six major pearl millet producing countries in Africa are Niger, Nigeria, Mali, Chad, Burkina Faso and Senegal. Average productivity in the region is 880 kg ha^− 1^ with Nigeria having the highest productivity. In Eastern and Southern Africa, pearl millet is cultivated on about 2 million ha, with productivity ranging from 800 kg ha^− 1^ to 920 kg ha^− 1^ (Bhagavatula et al., 2013).

In India 75% of total area under millets is occupied by pearl millet. Current total area under the crop is about 9 million ha with an average production of 7.68 million tons. The Rajasthan state, north and central Maharashtra and northern Karnataka together account for 72% of pearl millet area in India. Since late 1980s the yield levels have increased due to extensive use of improved varieties/hybrids of pearl millet. The yield levels are the highest (> 1 t ha^− 1^) in the semi-arid temperate regions and the lowest (0.5 t ha^− 1^) in the arid regions (Bhagavatula et al., 2013).

Low and erratic rainfall, high temperatures, poor soil fertility among the abiotic stresses and downy mildew disease and widespread *striga* infestation among biotic stresses, are the major constraints to the production of pearl millet. These constraints cause low and highly variable yields both in Asia and Africa. In addition to these constraints, the projected climate changes in the most arid and semiarid tropical regions will adversely impact the crop yields, thus challenging the food security in these regions (IPCC, 2007, Fischer et al., 2005, Howden et al., 2007). Changes in rainfall coupled with a rise in temperature may also reduce the length of the growing period (LGP) in the dry tropical regions (Cooper et al., 2009). Therefore, it will be important that the maturity duration of crops match the LGP in future for higher and stable yields.

The optimum range of air temperature for vegetative growth of pearl millet is 33 to 34 °C (Ong and Monteith, 1985). Fussell et al. (1980) reported that high temperatures (33/28 °C day/night) during vegetative, stem elongation and grain development phases decrease grain yield by reducing basal tillers, number of grains per inflorescence and single-grain weight. In the arid and semi-arid tropics the mean crop-season temperatures are already in the higher range for pearl millet production. Any further increase in temperature due to climate change will significantly reduce crop yields. However, increased CO_2_ concentration in the atmosphere could have beneficial effects on crop growth, and could partially negate the detrimental effects of rising temperatures depending on the degree of the temperature rise, and the extent of crop transpiration reductions under elevated CO_2_.

Knox et al. (2012), using meta-analysis of large number of publications, reported 10% reduction in yield of millet across Africa with climate change by 2050. Sultan et al. (2013) assessed the impacts of climate change on millet and sorghum yields in the Sudanian and Sahelian savannas of West Africa. They created multiple possible climate scenarios (thirty five) by combining precipitation changes from − 20% to 20% and temperature changes from + 0 to + 6 °C. Out of 35 scenarios tested 31 scenarios showed a negative impact on yield, up to − 41% (+ 6 °C/− 20% rainfall). They also concluded that changes in rainfall cannot counter the negative impacts caused by temperature rise when warming exceeds 2 °C. The Sudanian region appears to be more vulnerable as the probability of yield reduction appeared to be greater than in the Sahelian region. Their simulations also showed that the traditional millet and sorghum varieties used by the local farmers which are photoperiod-sensitive were more resilient to future climate conditions than the high yield potential modern cultivars.

Along with the development of new agronomic technologies, more changes in climate will require greater efforts to develop cultivars that are tolerant to drought, high temperatures and improved response to rising CO_2_ (Hammer et al., 2002, Boote et al., 2011). Because of the regional variations in climate change, there is a need for improved crop varieties, management practices and cropping systems that are specific to local conditions to adapt to the future climates. Attempts are already being made by plant breeders to identify key plant traits responsible for improved performance under climate change. At this juncture an assessment of benefits of incorporating such traits in pearl millet for the target environments would be useful for making appropriate research investments. Using weather, soil and agronomic management data, crop simulation models can be used to assess crop growth and yields with new technologies and study their yield advantages for target locations (Boote et al., 2001). Since these crop models incorporate genetic traits representing parameters, they can be used to propose genetic improvement of crops or to assess the possible benefits of new or modified plant traits on crop performance in a target environment (Landivar et al., 1983, Boote and Tollenaar, 1994, Boote et al., 2001, Boote, 2011, Yin et al., 1999, Hammer et al., 1996, Hammer et al., 2002, Hammer et al., 2005, Hammer et al., 2010, Tardieu, 2003, White and Hoogenboom, 2003, Messina et al., 2006, Suriharn et al., 2011, Singh et al., 2012, Singh et al., 2014a, Singh et al., 2014b, Singh et al., 2014c). Significant progress has been made on understanding the crop responses to climate change factors using simulation models and, these models provide an excellent opportunity to assess the benefits of genetic improvement of crops under climate change. Thus crop models provide a broader picture of impacts of climate change on crop performance. However, most crop models at current state of development do not simulate the impacts of pests and diseases and direct and indirect effects of extreme weather events such as high intensity rainfall storms and extended water-logging. As of today the authors of this paper have not come across any studies in the literature on use of pearl millet modeling framework to hypothesize genetic improvement of the crop to achieve higher yields, especially under climate change scenarios.

The objectives of this study were: 1) to assess the climate change impacts on the productivity of pearl millet at selected locations in West Africa and India; and 2) to assess the yield gains in pearl millet thorough genetic improvement, especially for drought and heat tolerance, for adapting to climate change in the pearl millet growing regions of West Africa and India.

## 2 Materials and methods

### 2.1 Study locations

The present study was carried out in two locations in West Africa and six locations in India. The six locations in India included four locations in the north-western zone (Hisar, Jaipur, Jodhpur and Bikaner) and two locations in the south-central zone (Aurangabad and Bijapur). In West Africa the two locations selected were Sadore (Niger) and Cinzana (Mali). The Hisar, Jodhpur and Bikaner locations in India are in the arid tropical climate and remaining five sites in India and West Africa are in the semi-arid tropical climate. At these locations pearl millet is extensively grown by farmers in their production systems. The soil and growing-season climatic characteristics of the locations are given in Table 1.

**Table 1:** Location, soil and climatic characteristics of selected sites in India and West Africa.

	India						West Africa	
	Hisar	Jaipur	Jodhpur	Bikaner	Aurangabad	Bijapur	Sadore	Cinzana
(a) Location								
Latitude (°)	29.15	26.91	26.24	28.02	19.88	16.82	13.25	13.25
Longitude (°)	75.72	75.79	73.02	73.32	75.34	75.71	2.3	− 5.96
Elevation (m)	221	100	514	223	282	404	300	280

(b) Soil								
Soil depth (cm)	168	80	169	130	120	176	210	180
EWHC (mm)^a^	195	72	202	196	139	198	167	117

(c) Climate (June to October)								
Mean max. temperature (°C)	36.0	35.4	36.5	38.5	31.1	31.6	35.3	32.7
Mean min. temperature(°C)	23.4	24.4	25.1	26.2	21.4	21.9	23.6	23.3
Mean temperature (°C)	29.7	29.9	30.8	32.4	26.2	26.8	29.5	28
Growing season rainfall (mm)	348	507	306	241	693	512	512	624
PET (mm)^b^	866	813	879	986	683	645	855	803

a Extractable water holding capacity of soil.

b Potential evapotranspiration.

### 2.2 The pearl millet model

We used DSSAT v4.6.10 (Decision Support System for Agro-technology Transfer, version 4.6.10) (Hoogenboom et al., 2014) as the starting point; however the existing CSM-CERES-pearl millet model (default version) was modified to assess the impact of climate change and plant traits on the productivity of pearl millet. The improvements made in the millet model are described in Section 2.5. This improved version of the model will be part of the next version of DSSAT. The model simulates crop growth and development (vegetative and reproductive) processes and dynamics of water, nitrogen and carbon in the soil. These processes have been described in detail by Ritchie et al. (1998) and Ritchie (1998). Jones et al. (2003) describe the minimum soil, weather, management and site data needed as input to the model to simulate a crop. The model also needs crop-specific parameters and cultivar-specific parameters or genetic coefficients (GCs) to simulate growth and yield of a pearl millet cultivar.

### 2.3 Baseline and projected weather data

Thirty-years (1980–2009) of observed daily weather data for the Indian locations were obtained from the India Meteorology Department, Pune, India, or downloaded from the NOAA site, except for the Bijapur site where only 25 years (1983–2007) data were available. Weather data for Sadore (1983–2008) and Cinzana (1983–2010) were obtained from the research stations of ICRISAT at Sadore (Niger) and Bamako (Mali), respectively. The baseline weather data of locations were checked for quality and the anomalous values, if found, were corrected using the bias-corrected AgMERRA data (Ruane et al., 2015). Future climate projections were created by utilizing “delta” approach, in which mean monthly climate changes from baseline under RCP 8.5 for near and mid-century period were applied to the daily baseline weather as per the mohc_hadgem2_es GCM model. Temperature changes were added to the baseline temperature, whereas precipitation change factors were multiplied to the precipitation data of the locations. The future time scale weather series and the corresponding projected CO_2_ concentration, according to RCP 8.5, were used in all simulations to study the climate change impacts. Projected changes in monthly temperatures and rainfall from the baseline values for the locations are presented in Table 2.

**Table 2:** Baseline climates and projected “delta” temperature or percent rainfall changes in climate at the selected sites in India.

Month	Hisar		Jaipur		Jodhpur		Bikaner	
	Baseline	Projected	Baseline	Projected	Baseline	Projected	Baseline	Projected
	(1980–2009)	(2050)^a^	(1980–2009)	(2050)	(1980–2009)	(2050)	(1980–2009)	(2050)
	Max. temperature (°C)							
June–Oct	33.4–39.9	0.9–3.7	33.3–39.9	0.1–2.7	34.1–40.3	0.1–2.5	36.4–41.9	− 0.2–2.4

	Min. temperature (°C)							
June–Oct	16.2–26.4	2.0–5.5	19.8–27.6	1.3–3.4	20.3–28.4	1.9–3.9	20.5–29.3	1.2–4.2

	Rainfall (mm) % change							
June	59	13	53	3	33	6	38	− 23
July	118	52	181	21	128	− 1	108	− 25
August	94	64	182	31	103	34	56	32
September	66	5	60	62	37	47	29	45
October	11	− 29	32	− 72	5	− 63	10	− 80
Total	348		507		306		241	

Month	Aurangabad		Bijapur		Sadore		Cinzana	
	Baseline	Projected	Baseline	Projected	Baseline	Projected	Baseline	Projected
	(1980–2009)	(2050)	(1983–2007)	(2050)	(1983–2008)	(2050)	(1983–2010)	(2050)
	Max. temperature (°C)							
June–Oct	28.9–34.4	0.3–3.6	30.5–33.7	− 0.1–2.7	32.6–37.7	1.6–3.0	30.5–35.2	2.4–4.9

	Min. temperature (°C)							
June–Oct	18.6–23.4	2.1–3.8	20.9–22.9	1.7–2.6	22.5–25.5	2.9–6.5	22.5–24.8	3.0–4.1

	Rainfall (mm) % change							
June	133	3	100	− 8	80	22	89	20
July	151	43	66	30	134	13	194	− 10
August	173	1	99	2	182	− 5	209	14
September	167	4	133	45	100	15	111	42
October	69	− 31	114	1	16	6	22	84
Total	693		512		512		624	

a 2040–2069 averaging period.

### 2.4 Soils data

For the Indian locations, the soil profile data were obtained from the published soil series of India (Lal et al., 1994). Soil data for the Sadore and Cinzana locations were obtained from the ICRISAT Research Stations at Sadore (Niger) and Bamako (Mali), respectively. Using this basic soil profile information and the SBuild tool available in DSSAT, layer-wise data on soil profile characteristics needed for model execution were estimated for all the selected locations.

### 2.5 The model improvements

The default version of the CSM-CERES-Pearl millet model had certain deficiencies that limited adequate simulation of the yield response to agronomic management and environmental factors, especially high temperatures. Certain changes in model code and parameters were necessary to improve the model and make it responsive to agronomic and environmental factors. The model underestimated grain yields when the crop was grown at low plant density or quite wide row-spacing. The power coefficient in the equation determining light interception by the canopy (LIFAC) was decreased from 0.1 to 0.04 to improve the model response to plant population/row-spacing. The model also underestimated the yield when drought stressed during reproductive period. To overcome this, the direct water stress effects were taken out from the equations in the model estimating panicle weight (PANWT), thus relying only on drought effects to reduce LAI and crop photosynthesis, and indirectly reducing panicle/grain growth. The model underestimated the leaf area of tillers as compared to the leaf area of main stem, thus minimizing their relative contribution to the overall yield of millet crop. A new genetic coefficient GT (tiller leaf size coefficient) was introduced in the cultivar file and code was added to enhance contribution of tillers to the total leaf area of the plant. Under extended day length the model over estimated grain yield and harvest index compared to the observed data because the extended day-length favored both the total biomass and panicle growth. The reduction in grain yield with delayed sowing was too strong in the model and also not consistent with the reduction in total biomass produced. This led to investigation on how grain numbers were being set in the model and required some changes in the code. The original code had 100% dependency of grain number and initial panicle mass on total biomass at the time of grain-set; the new code was modified to use 50:50 effect of crop growth rate during the ISTAGE4 phase (end of leaf growth to end of panicle growth) as well as total biomass at end of ISTAGE4 (end of panicle growth) when grain number and initial panicle mass were set. A new genetic coefficient G5 (potential grain size) was introduced in the cultivar file to set seed size, which also affected seed numbers set (see Appendix I for the explanation of GCs used in the modified version of the millet model). Some temperature-related response functions such as leaf area expansion (RGLAI) were hard-wired in the original model code; thus for the modified model these were externalized in the species file to allow correctly setting the upper limits of temperature thresholds for these processes. The temperature-related functions for photosynthesis (PRFTC) and grain growth rate (RGFIL), while already in the species file, were modified in their parameterization. Also the original model did not have any provision to affect seed number set by high temperatures (RGSET) and thus the effects of heat tolerance in pearl millet could not be evaluated. A new function (RGSET) was added to simulate high temperature effects on seed-set of pearl millet based on the work of Gupta et al. (2015). All the temperature-related response functions with original and modified temperature thresholds are given in Appendix II.

Thus the modified version of the CSM-CERES-Pearl millet model responds to various climate change factors such as elevated temperatures, elevated CO_2_ concentrations and rainfall variability. In the model, high temperature influences growth and development by shortening the crop life cycle and reducing allocation of biomass to reproductive organs through decreased seed set and seed growth rate. Increased CO_2_ concentration in the atmosphere increases crop growth and biomass production through small increase in radiation use efficiency (RUE) and transpiration efficiency. The effects of these two processes on pearl millet are simulated in the same way as for the sorghum crop, which simulations were described in detail by Singh et al. (2014c). Thus the pearl millet model has the ability to simulate the climate change impacts on growth, development and yield of pearl millet.

### 2.6 Model calibration

ICMH 356 and Sharda were considered as the most suitable baseline cultivars for Indian locations. Depending upon location and season, ICMH 356 takes 75 to 82 days to reach maturity, whereas Sharda takes 78 to 86 days to maturity. In the All India Coordinated Pearl Millet Improvement Project (AICPMIP) trials, these cultivars were used as national checks in the multi-location trials in India. ICMH 356 was used as a check in the north-western states (Punjab, Haryana, Rajasthan and Gujarat) and at some locations in the southern states (Andhra Pradesh, Karnataka and Tamil Nadu). Sharda was used as check in the central and southern states (Maharashtra, Karnataka, Andhra Pradesh and Tamil Nadu) of India. To determine GCs of these baseline cultivars, the Advanced Hybrid Trials data (1995–2012) of AICPMIP were used (AICPMIP, 1995–2012). The selected locations for ICMH 356 were Ludhiana, Delhi, Gwalior, Hisar, Bawal, Bikaner, Jaipur, Jodhpur, Anand and Anantapur. For Sharda the selected locations were Anantapur, Palem, Bijapur, Aurangabad, Buldana, Dhule, Mandore and Coimbatore. The available agronomic data included sowing date, harvest date, NPK applications and scheduling of irrigations to the crop. The observed data available from the variety trials were anthesis and maturity dates, seed size, and grain and fodder yields. For the drier locations in the north-western zone (Rajasthan and Haryana, rainfall < 450 mm during the growing season), plant population was 13 plants m^− 2^ with a row spacing of 60 cm. Across locations and seasons, nutrient management ranged from 40 to 80 kg N and 20 to 40 kg P ha^− 1^. All the P and half the N dose was applied at the time of sowing and the remaining N at 30 days after sowing. For the wetter locations in the north-western and south-central zones (rainfall > 450 mm during the growing season) the plant population was 18 plants m^− 2^ with a row spacing of 45 cm. Whenever the total N application in a season exceeded 80 kg ha^− 1^, the extra amount was applied at 50 days after sowing. GCs of pearl millet comprise those of phenology (P1, P2O, P2R and P5) and growth coefficients (G1, G4 and GT). These coefficients were determined by several iterations of the model such that the simulated data on phenology and crop yields matched the observed data across locations for a given pearl millet cultivar. Since the phenology of pearl millet is similar to that of sorghum, the iteration process followed to determine its phenology coefficients was the same as for sorghum (Singh et al., 2014c). Crop growth coefficients affecting total biomass production and its partitioning to panicle and seeds and crop canopy leaf area expansion were set by adjusting the scales for relative leaf size on main stem (G1), partitioning of assimilates to the panicle (G4) and relative leaf size on tillers (GT). SLPF (soil limited photosynthesis factor as given in the SOIL.SOL file) was also set to match the simulated yields with the observed data recorded across seasons for a site. The SLPF values used were 0.69 for Delhi, 0.95 for Ludhiana, 0.80 for Hisar, 0.91 for Bawal, 0.92 for Anand, 0.70 for Jaipur, 0.70 for Jodhpur, 0.82 for Bikaner, 0.76 for Aurangabad, 0.81 for Bijapur, 0.80 for Buldana, 0.78 for Dhule, 0.84 for Mandore, 0.65 for Anantapur, 0.74 for Palem and 0.77 for Coimbatore. Since the soil and weather data used for simulation did not belong, in a spatially exact sense, to the trial locations and since the records of crop agronomy and observed crop data had certain deficiencies, we calibrated the GCs of cultivars such that the mean, maximum and minimum grain yields simulated by the model were mostly within 15% of the reported mean, maximum and minimum grain yields for the locations. Thus, the rain-fed potential yield and the minimum yield were primarily obtained under drought stress and the mean yield for each site was simulated accurately.

For the West African locations the baseline cultivar used was CIVT. This cultivar is of medium duration and took about 105 to 112 days to mature at the selected target locations. All the growth, development and yield data used in this study for the CIVT cultivar were generated in field trials conducted at ICRISAT Research Centre at Sadore, Niamey, in Niger. The 1986 and 1987 seasons data (Sivakumar, 1990) and the 2005 season data (Akponikpe, 2008) were used for model calibration of GCs of the CIVT cultivar. These trials consisted of two to four sowing dates with some supplemental irrigation during the dry periods. Data were collected on 50% flowering, days to maturity, total biomass and grain yield. The model calibration procedure was the same as described above. The 1984, 1985, 1986 and 1987 seasons data (Sivakumar and Salaam, 1999) and 1995 season data (Akponikpe et al., 2008) were used for model evaluation. In these trials the crop was grown rainfed at different levels of N (0 to 60 kg ha^− 1^) and P (0 to 20 kg ha^− 1^) application in different years through chemical or organic sources. The crop in all these trials was grown in hills 1 m × 1 m apart and 3 plants per hill to give a plant population of 3 or 3.3 plants per m^2^. In the experiments where organic sources of nutrients were applied along with mineral fertilizers, the Century (Parton) method was used for the simulation of crop growth and yields; whereas where only mineral fertilizers were applied the CERES (Godwin) method was used. The SLPF values of the soils used for all the simulations were 0.55 each for Sadore and Cinzana, except for the experimental site during the 1995 season at Sadore when SLPF of 0.45 was used.

### 2.7 Virtual cultivars development

#### 2.7.1 Crop maturity duration and yield potential

For each baseline cultivar three crop maturity durations were identified for developing virtual cultivars – baseline (no change in the GCs), 10% shorter and 10% longer maturity cultivars. The procedure for making changes in crop maturity duration has been described in Singh et al. (2014c). To increase the yield potential of cultivars, the coefficients G1, G4 and GT in the cultivar file and radiation use efficiency (RUE) in the ecotype file (*.ECO) were increased by 10% each. These procedures resulted in six virtual cultivars, namely, baseline, 10% short, 10% long, baseline + yield potential, 10% short + yield potential and 10% long + yield potential, to which the drought and heat tolerance traits were incorporated virtually.

#### 2.7.2 Drought and heat tolerance

Changes were made in WR (relative root length density distribution with soil depth) and LL (lower limit of soil water availability) for each soil layer given in the soil file (*.SOL) to enhance drought tolerance of cultivars. The procedure for creating drought tolerant virtual cultivars has been described in detail by Singh et al., 2014b, Singh et al., 2014c. Heat tolerant pearl millet virtual cultivar was created by increasing the two upper threshold values of each RGFIL (relative grain filling rate with temperature) and RGSET (relative grain set rate with temperature) located in the species file (*.SPE) by 2 °C. In case of RGFIL the upper optimum temperature threshold (TOP2) value was increased from 27 to 29 °C and the damaging (failure) temperature threshold (TMAX) value increased from 60 to 62 °C. In case of RGSET the TOP2 was increased from 33 to 35 °C and the TMAX from 39 to 41 °C (see Appendix II for details).

Heat and drought tolerance traits were evaluated for the base + yield potential virtual cultivar for the locations, unless the other virtual cultivar was significantly high yielding. Thus considering their mean yields under baseline climate and climate change, the base + yield potential virtual cultivar was evaluated for the Jaipur, Jodhpur, Bikaner, Sadore and Cinzana locations; whereas the 10% long + yield potential cultivar for the Hisar, Aurangabad and Bijapur locations.

### 2.8 Simulation runs

The modified CSM-CERES-Pearl millet simulation model coupled with the seasonal analysis tool available in DSSAT v4.6.1.0 was used to simulate climate change impacts on pearl millet productivity. For each study location, we carried out simulations for both baseline and the projected climate change (Temperature + CO_2_ + Rainfall) by 2030 and 2050. The atmospheric CO_2_ concentration used were 380 ppm, 432 ppm and 571 ppm for the baseline, 2030 and 2050 climate projections, respectively (Rosenzweig et al., 2015). For evaluating genetic traits, simulations were carried out for the baseline and 2050 climate change projections only.

Each year simulations were initiated on 1st January when the soil profile was assumed to be at lower limit of water availability (LL). Each year the sowing window was first week of July to end of August for Hisar, Jaipur, Jodhpur and Bikaner locations and third week of June to last week of August for the Aurangabad and Bijapur locations. The simulated crop was sown on the day within the sowing window when the soil moisture had reached at least 40% of the extractable water-holding capacity in the top 30-cm soil. For the drier locations (Hisar, Jaipur, Jodhpur and Bikaner) a plant population of 13 plants per m^2^ with a row-to-row spacing of 60 cm was considered for simulating the pearl millet yield. At sowing 18 kg N ha^− 1^ were applied as di-ammonium phosphate and the second dose of 22 kg N ha^− 1^ applied as urea at 30 days after sowing. For the wetter locations (Aurangabad and Bijapur), plant population considered was 18 plants per m^2^ with a row-to-row spacing of 45 cm. At sowing 30 kg N ha^− 1^ were applied as mixture of di-ammonium phosphate and urea. Additional dose of 30 kg N ha^− 1^ was applied as urea at 30 days after sowing. For the Sadore and Cinzana locations the plant population considered was 3 plants m^− 2^. At sowing 15 kg N ha^− 1^ was applied at sowing at both the locations; the second dose of nitrogen was 15 kg N ha^− 1^ at Sadore and 25 kg N ha^− 1^ at Cinzana applied at 20 days after sowing; whereas the third dose of nitrogen was 30 kg N ha^− 1^ at Sadore and 40 kg N ha^− 1^ at Cinzana applied at 45 days after sowing. As the study primarily focused on the climate change impacts, the P-module in the millet model was turned off and did not respond to any P application. The SLPF (soil-limited photosynthesis factor) values for the selected locations were the same as used for model calibration for each site (see Section 2.6). For each climate scenario (baseline and climate change) and for evaluating the genetic traits the simulations were carried out for 30 years each for Hisar, Jodhpur, Jaipur, Bikaner and Aurangabad; whereas for Bijapur, Sadore and Cinzana these were done for 25, 26 and 28 years, respectively. As the pearl millet model does not simulate the effects of biotic stresses, the crop was considered free from pests and diseases. All the simulated data were analyzed using GenStat software by following the analysis of variance (ANOVA) method (Payne et al., 2009). To compare the treatments, RBD (Randomised complete block design) was followed and the LSD at 5% level of probability was calculated to compare the treatments. Years were considered as replications (blocks), as the pearl millet yield of a given treatment in one year was not affected by the prior year (no carry-over effect of soil water from one year to another).

## 3 Results

### 3.1 Model validation

**Fig. 1:**
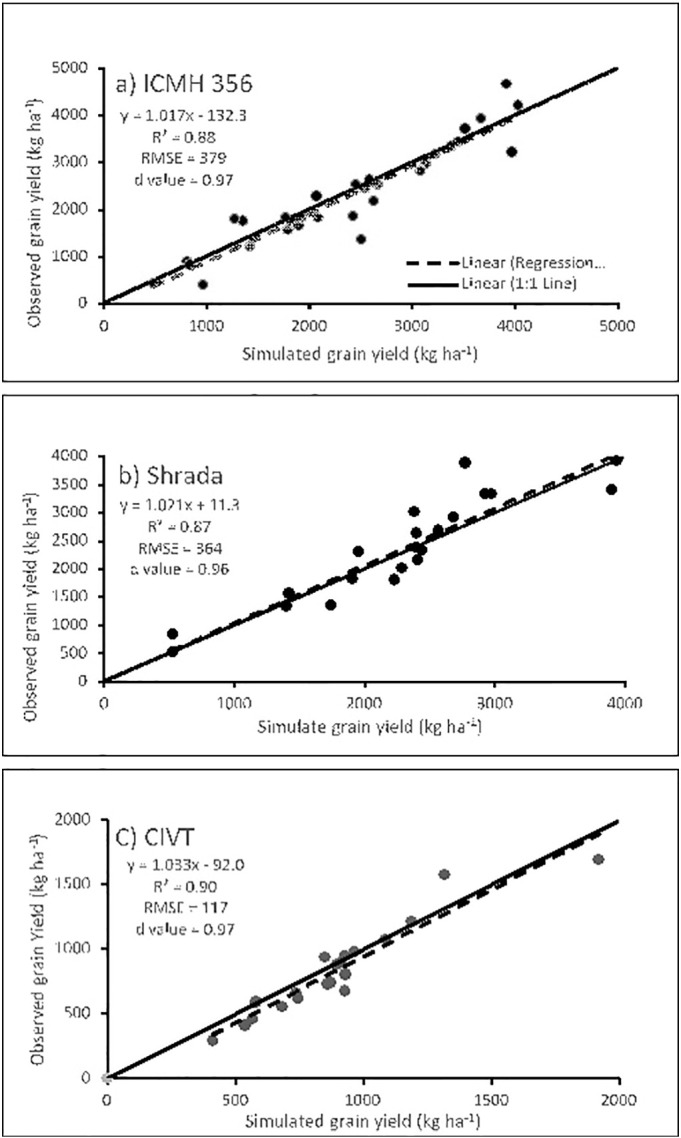
Relationship of simulated seed yield with the observed yield across sites in India for cultivars (a) ICMH 356, (b) Sharda and (c) CIVT.

Simulated grain yields of baseline cultivars were significantly correlated with observed data of the three cultivars (Fig. 1). The d-value, an index of model predictability (Willmott, 1982), was 0.97 for ICMH 356, 0.96 for Sharda and 0.97 for CIVT. These results confirm that the modified model can accurately simulate the growth and yield of pearl millet for the millet-growing regions of India and West Africa.

### 3.2 Impact of climate change on pearl millet yield

At Hisar and Jaipur the cultivar ICMH 356 on average produced 1019 kg ha^− 1^ and 1405 kg ha^− 1^ of grain yield, respectively (Table 3). Climate change by 2030 significantly (p < 0.05) increased the grain yield at Hisar by 34%, mostly because of increased rainfall at that location, but not at Jaipur. Overall effect of climate change factors (temp. + CO_2_ + rain) by 2050 was 8% increase in yield at Hisar, which was statistically non-significant (p < 0.05). At Jaipur, however, the yield significantly (p < 0.05) decreased by 7% as compared to the baseline yield. The decrease in yield at Jaipur could be due increased crop water stress on the light textured shallow soil with the increase in temperature in spite of increase in rainfall at this site.

**Table 3:** Impact of climate change (temperature + CO_2_ + rainfall) on pearl millet grain yield (kg ha^− 1^) at selected sites in India and West Africa.

Time slice	Climate scenario	Hisar		Jaipur		Jodhpur		Bikaner	
		Yield ± s.d	% change	Yield ± s.d	% change	Yield ± s.d	% change	Yield ± s.d	% change
Baseline	Baseline	1019 ± 946		1405 ± 736		1357 ± 1108		518 ± 472	
2030	Temp. + CO_2_ + rain	1367 ± 925	34	1418 ± 643	1	1663 ± 1159	23	627 ± 704	21
2050	Temp. + CO_2_ + rain	1105 ± 810	8	1303 ± 531	− 7	1531 ± 1168	13	544 ± 651	5
LSD (0.05)^a^		102		86		125		75	

		Aurangabad		Bijapur		Sadore		Cinzana	
		Yield	% change	Yield	% change	Yield	% change	Yield	% change
Baseline	Baseline	2241 ± 971		1795 ± 610		1188 ± 498		1321 ± 447	
2030	Temp. + CO_2_ + rain	2230 ± 744	− 1	1865 ± 645	4	1095 ± 456	− 8	1470 ± 294	11
2050	Temp. + CO_2_ + rain	1925 ± 727	− 14	1729 ± 538	− 4	1018 ± 395	− 14	1375 ± 289	4
LSD (0.05)^a^		117		183		76		114	

s.d = Standard deviation; temp. = temperature; CO_2_ = carbon dioxide; R = rainfall; % change = percent change in yield with respect to the baseline yield;

a Least significant difference at 5% level of probability to compare yields within the same column.

At Jodhpur and Bikaner, the baseline cultivar ICMH 356 on average produced 1357 kg ha^− 1^ and 518 kg ha^− 1^ of grain yield under baseline climate, respectively (Table 3). Overall effect of climate change (temp. + CO_2_ + rain) by 2030 was 23% increase in yield at Jodhpur and 21% at Bikaner, indicating significant (p < 0.05) and positive effect of increase in rainfall on yields at the two locations. Climate change by 2050 increased the grain yield by 13% over the baseline yield at Jodhpur, which was statistically significant (p < 0.05), while there was a non-significant increase of 5% at Bikaner.

For the South-Central zone the growth and yield of cultivar Sharda was simulated. Under baseline climate on average it produced 2241 kg ha^− 1^ of grain yield at Aurangabad; whereas at Bijapur it produced 1795 kg ha^− 1^ (Table 3). Overall effect of climate change (temp. + CO_2_ + rain) by 2030 was 1% decrease in yield at Aurangabad and 4% increase at Bijapur, which were non-significant (p < 0.05) changes in yields at both locations. Climate change by 2050 decreased the yield by 14% and 4% at Aurangabad and Bijapur, respectively, which was statistically significant (p < 0.05) at Aurangabad, but not at Bijapur. The larger decrease in yield at Aurangabad could be attributed to low water holding capacity of soil and the least increase in rainfall after July as compared to Bijapur and other selected locations in India.

At Sadore, the cultivar CIVT on average produced 1188 kg ha^− 1^ of grain yield under baseline climate; whereas at Cinzana it produced 1321 kg ha^− 1^ (Table 3). Overall effect of climate change (temp. + CO_2_ + rain) by 2030 was 8% decrease in yield at Sadore and 11% increase at Cinzana. These changes in yields at both of these locations were statistically significant (p < 0.05) as compared to the baseline yields at the respective locations. Overall effect of climate change factors (temperature + CO_2_ + rainfall) by 2050 was significant (p < 0.05) decrease in yield at Sadore (− 14%) and non-significant increase at Cinzana (4%). In spite of some reduction in rainfall during July at Cinzana, the increasing trend in yield at Cinzana could be attributed to overall better moisture regime with climate change at Cinzana than at Sadore.

Climate change at most sites, except Jodhpur and Bikaner, decreased the standard deviation in yields associated with mean yields under climate change. This may be attributed to increase in rainfall at the sites in future due to climate change. Jodhpur and Bikaner are in more arid climate where climate change increased the standard deviation in yields, which is one of the measures of uncertainty in yields.

### 3.3 Pearl millet response to genetic traits in India

At Hisar under baseline climate, the cultivar ICMH 356 matured in 75 days and on average produced 1019 kg ha^− 1^ of grain yield (Table 4). Under baseline climate, the yield of 10% shorter duration cultivar decreased by 19%, whereas increasing the duration by 10% increased the yield by 17% (Table 4). Yield potential traits increased the yield by 21% to 26%, the highest increase being with the shorter duration cultivar and the lowest with the 10% longer duration cultivar. However, the maximum yield (1449 kg ha^− 1^) was simulated for the 10% longer duration cultivar having yield potential traits incorporated. Under climate change, the pattern of yield response to crop maturity duration and yield potential traits was similar to that simulated for the baseline climate. Thus, the longer duration cultivar, with and without yield potential traits, gave the highest yield under both the baseline and climate change scenarios. At Jaipur, the same cultivar matured in 76 days and on average produced 1405 kg ha^− 1^ grain yield under baseline climate (Table 4). Under both climate scenarios, the percent change in yield due to incorporation of yield potential and crop maturity traits followed the similar trend as for the Hisar site. However, the base + yield potential virtual cultivar had significantly (p < 0.05) higher yield compared to the baseline cultivar under both climates.

**Table 4:** Performance of virtual cultivars under baseline climate and climate change (temp. + CO_2_ + rain) by 2050 at Hisar and Jaipur sites in India. Grain yield in kg ha^− 1^.

Virtual cultivars	Baseline climate		Climate change 2050	
	Grain yield	% change	Grain yield	% change
	Hisar			
Baseline	1019	–	1105	–
10% short	822	− 19	907	− 18
10% long	1195	17	1294	17
Baseline + yield pot.	1259	24^a^	1416	28^a^
10% short + yield pot.	1036	26^a^	1182	30^a^
10% long + yield pot.	1449	21^a^	1622	25^a^
LSD (0.05)^b^	178		194	

10% long + yield pot.	1449	–	1622	–
10% long + yield pot. + drought tolerance (DT)	1567	8^c^	1761	9^c^
10% long + yield pot. + heat tolerance (HT)	1489	3^c^	1751	8^c^
10% long + yield pot. + DT + HT	1614	11^c^	1892	17^c^
LSD (0.05)^b^	54		64	

	Jaipur			
Baseline	1405		1303	
10% short	970	− 31	874	− 33
10% long	1492	6	1358	4
Baseline + yield pot.	1661	18^a^	1574	21^a^
10% short + yield pot.	1293	33^a^	1252	43^a^
10% long + yield pot.	1658	11^a^	1537	13^a^
LSD (0.05)^b^	220		185	

Baseline + yield pot.	1661		1575	
Baseline + yield pot. + drought tolerance (DT)	1796	8^c^	1683	7^c^
Baseline + yield pot. + heat tolerance (HT)	1732	4^c^	1672	6^c^
Baseline + yield pot. + DT + HT	1852	11^c^	1786	13^c^
LSD (0.05)^b^	38		32	

% change: percent yield gain of a virtual cultivar due to the trait as compared to the baseline cultivar yield unless otherwise indicated.

a Yield improvement compared to the cultivar with same crop maturity.

b Least significant difference at 5% level of probability to compare yields within the column above the LSD value.

c Yield improvement compared to the cultivar with same crop maturity and yield potential.

At Hisar the yield increased by 8% with drought tolerance under base climate and 9% under climate change (Table 4). Increase in yield due to heat tolerance was only 3% under baseline climate, which significantly (p < 0.05) increased up to 8% under climate change. Both drought and heat tolerance combined increased the yield by 11% under baseline climate and 17% under climate change. At Jaipur, drought tolerance increased the yield by 8% and 7% under baseline climate and climate change, respectively, which were significant (p < 0.05) increases (Table 4). The benefit in yield due to heat tolerance at Jaipur was only 4% under baseline climate and 6% under climate change, however, the yield increases were statistically significant (p < 0.05). Both drought and heat tolerance combined gave 11% yield increase under baseline climate and 13% in climate change conditions.

Jodhpur and Bikaner being warmer locations than Hisar and Jaipur, the baseline cultivar ICMH 356 matured a few days earlier than at Hisar and Jaipur and on average it produced 1357 kg ha^− 1^ of grain yield at Jodhpur and 518 kg ha^− 1^ at Bikaner under baseline climate (Table 5). At Jodhpur the grain yields of 10% shorter or 10% longer maturity cultivars were not significantly (p < 0.05) different from the baseline cultivars yield under baseline climate. Yield potential traits increased the yield of virtual cultivars by 15% to 18% with maximum yield gain for the 10% short duration cultivar. Under climate change, the yield of shorter duration cultivar decreased by 16%; whereas, the yield of longer duration cultivar increased by 6%, which was a non-significant (p < 0.05) increase in yield. Yield potential traits significantly (p < 0.05) increased the yield of virtual cultivars by 18% to 24% under climate change. At Bikaner, the changes in grain yield due to crop maturity or yield potential traits were non-significant under both climate scenarios, except for the longer duration cultivar with yield potential traits that produced significantly higher yield under baseline climate (Table 5).

**Table 5:** Performance of virtual cultivars under baseline climate and climate change (temp. + CO_2_ + rain) by 2050 at Jodhpur and Bikaner sites in India. Grain yield in kg ha^− 1^.

Virtual cultivars	Baseline climate		Climate change 2050	
	Grain yield	% change	Grain yield	% change
	Jodhpur			
Baseline	1357		1531	
10% short	1288	− 5	1286	− 16
10% long	1369	1	1620	6
Baseline + yield pot.	1592	17^a^	1832	20^a^
10% short + yield pot.	1519	18^a^	1597	24^a^
10% long + yield pot.	1577	15^a^	1909	18^a^
LSD (0.05)^b^	206		218	

Baseline + yield pot.	1592		1832	
Baseline + yield pot. + drought tolerance (DT)	1847	16^c^	1948	6^c^
Baseline + yield pot. + heat tolerance (HT)	1654	4^c^	1974	8^c^
Baseline + yield pot. + DT + HT	1909	20^c^	2089	14^c^
LSD (0.05)^b^	108		71	

	Bikaner			
Baseline	518		544	
10% short	456	− 12	504	− 7
10% long	583	12	542	0
Baseline + yield pot.	601	16^a^	647	19^a^
10% short + yield pot.	528	16^a^	601	19^a^
10% long + yield pot.	690	18^a^	650	20^a^
LSD (0.05)^b^	142		114	

Baseline + yield pot.	601		647	
Baseline + yield pot. + drought tolerance (DT)	717	19^c^	789	22^c^
Baseline + yield pot. + heat tolerance (HT)	673	12^c^	754	17^c^
Baseline + yield pot. + DT + HT	805	34^c^	919	42^c^
LSD (0.05)^b^	34		90	

% change: percent yield gain of a virtual cultivar due to the trait as compared to the baseline cultivar yield unless otherwise indicated.

a Yield improvement compared to the cultivar with same crop maturity.

b Least significant difference at 5% level of probability to compare yields within the column above the LSD value.

c Yield improvement compared to the cultivar with same crop maturity and yield potential.

At Jodhpur drought tolerance trait increased the yield by 16% under base climate and 6% under climate change (Table 5). Heat tolerance increased the yield by 4% under baseline climate and 8% in climate change conditions. Drought and heat tolerance together gave a yield advantage of 20% and 14% under baseline climate and climate change, respectively. At Bikaner the yield gain due to drought tolerance was 19% under baseline climate and 22% under climate change, which were the highest gains in yields in percentage terms in the respective climates due to drought tolerance among the Indian locations (Table 5). Bikaner was the warmest of the selected locations in India, thus the yield benefit due to heat tolerance in percentage terms was the highest, which was 12% and 17% under baseline climate and climate change, respectively. The combined benefit of drought and heat tolerance was also the highest at this site, which was 34% and 42%, respectively, for the two climates. At Jodhpur and Bikaner locations all the yield gains due to drought or heat tolerance were statistically significant (p < 0.05) under both the baseline and climate change scenarios.

At Aurangabad the cultivar Sharda took 85 days to mature under baseline climate and on average produced 2241 kg ha^− 1^ of grain yield; whereas at Bijapur it took 84 days to mature and produced 1795 kg ha^− 1^ of grain yield (Table 6). Reducing crop maturity by 10% decreased crop yield by 42%, whereas increasing crop maturity by 10% increased yield by 47%. Yield potential traits increased yield by 25% to 40% (Table 6). The highest increase in yield due to yield potential traits was with the 10% shorter duration cultivar and the lowest with the 10% longer duration cultivar. Under climate change, the yield of shorter duration cultivar decreased by 42% and that of longer duration increased by 46%. Yield potential traits increased the yield of virtual cultivars by 31% to 43%. As under baseline climate, the yield increase due to yield potential traits was the greatest with the shorter duration and the lowest with the longer duration cultivar. Under both climates, however, the maximum absolute yield was obtained for the longer maturity cultivar along with yield potential traits. At Bijapur, the crop maturity and yield potential traits increased the yield of virtual cultivars in way similar to the Aurangabad site under both the climates; however, the magnitude of absolute yields was different from those simulated for the Aurangabad site (Table 6). At both Aurangabad and Bijapur locations under baseline and climate change scenarios, the changes in crop yields due to crop maturity or yield potential traits were statistically significant (p < 0.05). Drought tolerance increased the yields by was 6% and 7% at Aurangabad under baseline climate and climate change, respectively (Table 6). Aurangabad being the coolest of all the selected locations, heat tolerance did not enhance the yields under both the climates. For the Bijapur site, the yield gain due to drought tolerance was 15% under baseline climate and 13% under climate change (Table 6). Like Aurangabad site, heat tolerance at this site had no beneficial effect on pearl millet yields under both the climate scenarios. However, the yield gains due to drought tolerance at Aurangabad and Bijapur were statistically significant (p < 0.05) under both baseline climate and climate change.

**Table 6:** Performance of virtual cultivars under baseline climate and climate change (temp. + CO_2_ + rain) by 2050 at Aurangabad and Bijapur sites in India. Grain yield in kg ha^− 1^.

Virtual cultivars	Baseline climate		Climate change 2050	
	Grain yield	% change	Grain yield	Grain yield
	Aurangabad			
Baseline	2241		1925	
10% short	1306	− 42	1113	− 42
10% long	3292	47	2814	46
Baseline + yield pot.	2964	32^a^	2641	37^a^
10% short + yield pot.	1831	40^a^	1588	43^a^
10% long + yield pot.	4101	25^a^	3698	31^a^
LSD (0.05)^a^	265		220	

10% long + yield pot.	4101		3698	
10% long + yield pot. + drought tolerance (DT)	4346	6^c^	3956	7^c^
10% long + yield pot. + heat tolerance (HT)	4107	0^c^	3737	1^c^
10% long + yield pot. + DT + HT	4352	6^c^	3999	8^c^
LSD (0.05)^b^	66		68	

	Bijapur			
Baseline	1795		1729	
10% short	1086	− 39	915	− 47
10% long	2628	46	2531	46
Baseline + yield pot.	2360	31^a^	2356	36^a^
10% short + yield pot.	1523	40^a^	1393	52^a^
10% long + yield pot.	3335	27^a^	3363	33^a^
LSD (0.05)^b^	216		188	

10% long + yield pot.	3335		3363	
10% long + yield pot. + drought tolerance (DT)	3848	15^c^	3808	13^c^
10% long + yield pot. + heat tolerance (HT)	3337	0^c^	3400	1^c^
10% long + yield pot. + DT + HT	3851	15^c^	3824	14^c^
LSD (0.05)^b^	109		131	

% change: percent yield gain of a virtual cultivar due to the trait as compared to the baseline cultivar yield unless otherwise indicated.

a Yield improvement compared to the cultivar with same crop maturity.

b Least significant difference at 5% level of probability to compare yields within the column above the LSD value.

c Yield improvement compared to the cultivar with same crop maturity and yield potential.

### 3.4 Pearl millet response to genetic traits in West Africa

At Sadore, cultivar CIVT took 105 days to mature and produced 1188 kg ha^− 1^ of grain yield under baseline climate (Table 7), whereas, at Cinzana it took 112 days to mature and produced 1321 kg ha^− 1^ of grain yield. Higher mean yield at Cinzana could be attributed to higher seasonal rainfall at Cinzana than at Sadore (Table 1). At Sadore under baseline climate, the yield of 10% shorter and 10% longer maturity cultivars decreased by 3% and 15%, respectively. Incorporating yield potential traits increased the yield of virtual cultivars by 11% to 14%. Under climate change, the yield of both shorter and longer maturity cultivars decreased and the decrease was significant (p < 0.05) for the shorter maturity cultivar (17%). Yield potential traits significantly (p < 0.05) increased the yield of virtual cultivars, which ranged from 12% to 21% with the greatest increase for the shorter maturity cultivar (Table 7). At Cinzana, the yield of shorter and longer duration cultivar decreased by 10% and 17%, respectively, under baseline climate. Yield potential traits increased the yield of virtual cultivars up to 10%, which was statistically non-significant (p < 0.05). Under climate change the grain yield of shorter maturity cultivar significantly (p < 0.05) decreased by 29% and that of longer maturity increased by 4%. Yield benefit of yield potential traits ranged from 10% to 21% across virtual cultivars. Under climate change the highest yield was simulated for the 10% longer maturity cultivar with yield potential traits (1582 kg ha^− 1^), but did not differ significantly (p < 0.05) from the baseline cultivar with yield potential traits (Table 7).

**Table 7:** Performance of virtual cultivars under baseline climate and climate change (temp. + CO_2_ + rain) by 2050 at Sadore and Cinzana sites in West Africa. Grain yield in kg ha^− 1^.

Virtual cultivars	Baseline climate		Climate change 2050	
	Grain yield	% change	Grain yield	Grain yield
	Sadore			
Baseline	1188		1018	
10% short	1150	− 3	845	− 17
10% long	1009	− 15	962	− 6
Baseline + yield pot.	1323	11^a^	1165	14^a^
10% short + yield pot.	1310	14^a^	1021	21^a^
10% long + yield pot.	1137	13^a^	1082	12^a^
LSD (0.05)^b^	153		109	

Baseline + yield pot.	1323		1165	
Baseline + yield pot. + drought tolerance (DT)	1723	30^c^	1361	17^c^
Baseline + yield pot. + heat tolerance (HT)	1393	5^c^	1321	13^c^
Baseline + yield pot. + DT + HT	1812	37^c^	1548	33^c^
LSD (0.05)^b^	55		42	

	Cinzana			
Baseline	1321		1375	
10% short	1187	− 10	972	− 29
10% long	1092	− 17	1434	4
Baseline + yield pot.	1435	9^a^	1548	13^a^
10% short + yield pot.	1304	10^a^	1173	21^a^
10% long + yield pot.	1206	10^a^	1582	10^a^
LSD (0.05)^b^	135		106	

Baseline + yield pot.	1435		1548	
Baseline + yield pot. + drought tolerance (DT)	1602	12^c^	1676	8^c^
Baseline + yield pot. + heat tolerance (HT)	1467	2^c^	1649	6^c^
Baseline + yield pot. + DT + HT	1638	14^c^	1784	15^c^
LSD (0.05)^b^	37		26	

% change: percent yield gain of a virtual cultivar due to the trait as compared to the baseline cultivar yield unless otherwise indicated.

a Yield improvement compared to the cultivar with same crop maturity.

b Least significant difference at 5% level of probability to compare yields within the column above the LSD value.

c Yield improvement compared to the cultivar with same crop maturity and yield potential.

At Sadore, drought tolerance increased the yield by 30% under baseline climate and 17% under climate change. Whereas, at the Cinzana, the yield gains due to this trait were 12% under baseline climate and 8% under climate change (Table 7). Lesser benefit due to drought tolerance under climate change at Cinzana is attributed to better rainfall regime during the season under climate change than at Sadore. Sadore being the warmer site than Cinzana, the yield gain due to heat tolerance was significantly (p < 0.05) greater at Sadore (5%) than at Cinzana (2%) under baseline climate. However, under climate change the yield benefit due to heat tolerance significantly (p < 0.05) increased at both locations (13% at Sadore and 6% at Cinzana). Both drought and heat tolerance combined had additive effects on yield under both baseline and climate change scenarios at the two locations in West Africa.

## 4 Discussion

Using the modified CSM-CERES-Pearl Millet model, we evaluated the genetic traits of pearl millet for adaptation to climate change at selected locations in West Africa and India. The study revealed that in the higher rainfall environments of Aurangabad and Bijapur, the potential yield gains with the 10% longer maturity cultivar are to the extent of 47% as compared to the baseline cultivar (Sharda) under current climate and climate change by midcentury (Table 6). At other locations (Hisar, Jaipur, Jodhpur and Bikaner) although the yields increased with 10% longer maturity, they were statistically non-significant (p < 0.05) in the both present and future climates. So the baseline cultivar life cycle duration (ICMH 356) remained the highest yielding at the four locations under both climate regimes, possibly because of better fit to the rainfall patterns of those sites which minimized benefit of the longer maturity types. Similarly, at Sadore and Cinzana under base climate the yields were higher for the baseline cultivar (CIVT) as compared to the yields with the 10% shorter or longer maturity cultivar (Table 7). Under climate change the baseline cultivar gave the highest yield at Sadore; however, at Cinzana a 4% increase in yield was simulated with 10% longer maturity cultivar, which was statistically non-significant (p < 0.05). For the favorable rainfall conditions of future, the cultivars that are of longer maturity in current climate will generally be more suitable as the warmer climate typically shortens the life cycle and longer maturity cultivars will compensate for these conditions and produce higher yields than the default baseline cultivars. Identifying a proper cultivar according to length of growing period is one of the best ways to tackle climate change impacts as sufficient genetic diversity exists in pearl millet maturity groups (Rai et al., 1999). This would minimize drought and heat stress during the crop life cycle and the available seasonal resources would be fully utilized.

In addition to increase or decrease in crop duration, altering yield potential-controlling genetic traits (RUE, G1, G4 and GT) were also tried as adaptation options across locations in India and Africa. Incorporating yield potential traits in the longer maturity cultivar increased the yield up to 25% at Hisar, 13% at Jaipur, 18% at Jodhpur, 20% at Bikaner, 31% at Aurangabad and 33% at Bijapur under climate change. However, the yield improvements for the baseline and shorter maturity cultivars with yield potential traits were even larger. At Sadore under climate change, the yield of the baseline cultivar was the highest (1018 kg ha^− 1^), which further increased by 14% by incorporating yield potential traits (Table 7); whereas, at Cinzana under climate change the grain yield of the high yielding longer duration cultivar was further enhanced by 10% by incorporating yield potential traits. Yield gains associated with changing yield potential traits virtually were mainly due to changes in sources and sink size. Rai et al. (1999) and Ong and Monteith (1985) identified several genotypes in pearl millet having different source and sink sizes. Based on our study it was clearly evident that genotypes having higher RUE (source) and G1, G4 and GT (sink size) will produce higher yields under climate change conditions.

Incorporating drought tolerance in pearl millet by increasing density of roots in the subsoil and, therefore, greater soil water extraction with depth was tried as another way to adapt to climate change. In India under baseline climate, the yield improvements due to drought tolerance traits were the highest at Bikaner followed by Jodhpur, Bijapur, Hisar, Jaipur and Aurangabad; while in West Africa the yield gains were higher at Sadore than at Cinzana. The reason for differential response was mainly due to differences in soil water availability at the target locations as determined by the water holding capacity of soils and the amount and distribution of rainfall. Except for the Bikaner site, the percent yield gains due to drought tolerance of virtual cultivars under climate change were either the same or somewhat less than those simulated under baseline climate, in part because of increasing rainfall under climate change in much of India. The locations where rainfall is projected to decrease during mid-century period, the drought tolerant cultivars were found to be more promising adaptation option. Further, even at locations where the rainfall is projected to increase in future, the drought-tolerant virtual cultivars were still found to be suitable as the crops will still suffer from drought due to increased water demand caused by higher air temperatures as projected during mid-century. In addition to other multiple mechanisms, increasing root length density (RLD) and rooting depth in the subsoil are suggested as the prominent mechanisms for drought tolerance and higher yields in pearl millet and other dryland crops (Vadez et al., 2012). Genetic variation in root traits of pearl millet exists, which can be used for breeding drought tolerant cultivars (Kusaka et al., 2005, Vadez et al., 2012). Increased RLD in subsoil, thus greater soil water extraction with depth, also enhances water use efficiency (yield/evapotranspiration) of the crop because most of the additional water uptake from the subsoil is lost as transpiration. Thus, the approach used in the model to simulate drought tolerance of virtual cultivars is appropriate. However, there is a chance that drought-tolerant cultivars may also result in greater extraction of subsoil water which will impact the following crop in a cropping system. Hence, the analysis on system prospective to study the carry-over effect of water to the next crop is needed.

In addition to drought, high temperatures during the cropping season also adversely affect grain yields in current climate at most locations and will be even more damaging under climate change with increasing temperatures. In current climate, the benefit of incorporating up to 2 °C greater heat tolerance had maximum yield advantage at Bikaner (12%) followed by Sadore, Jodhpur, Jaipur, Hisar and Cinzana. Because of current low temperatures at Aurangabad and Bijapur, heat tolerance trait had no effect on grain yield there. However, under climate change the benefit of incorporating heat tolerance further increased at all the locations, except at Aurangabad and Bijapur. The maximum benefit of heat tolerance in terms of yield increase was 17% at Bikaner, followed by 13% at Sadore, 8% each at Jodhpur and Hisar and 6% each at Jaipur and Cinzana. Heat tolerance exists among pearl millet genotypes and this has been amply demonstrated by Gupta et al. (2015) in field trials conducted during spring-summer season (March to May) in India. Thus heat tolerant germplasm could be used to breed new varieties to enhance heat tolerance in pearl millet under future climate change conditions. Combining drought tolerance with heat tolerance in a virtual cultivar further enhanced the yields and the effects were mostly additive in both current and future climates.

The simulation study investigated the role of genetic improvement of pearl millet for adapting to climate change in future. Because the climate changes are projected to be small in the near-future, short-term agronomic adjustments (such as changing the sowing date, fertilizer management, water management etc.) may be more readily useful than genetic options for adapting to climate change (Easterling, 1996, Howden et al., 2007). Under more adverse climate change, a combination of both improved agronomic and genetic options will be needed for adapting to climate. For each target site these options would need to be prioritized, in terms of yield or economic advantage, for greater adoption by the farmers under climate change. This study focused on the major plant traits that are believed to be most useful in adapting pearl millet to climate change in the arid and semi-arid tropical environments in India and West Africa. There may be some other site-specific plant traits, such as resistance to insect pests and plant diseases, useful for adapting to climate change that have not been considered here. Additionally, adaptation to extreme weather events beyond drought and heat (such as water-logging and wind) were not considered here. Overall, this study has broad application for adapting pearl millet to climate change.

## 5 Conclusions

This study focused on the major crop productivity constraints associated with climate change in future in the millet growing environments in India and West Africa. The study revealed that under climate change conditions Hisar, Jaipur, Jodhpur, Bikaner, Sadore and Cinzana will need baseline or slightly longer duration cultivars along with drought and heat tolerance traits. However, Aurangabad and Bijapur will benefit from longer duration cultivars with only drought tolerance for higher yields in future climates. Yield potential traits will be useful at all the locations under climate change. As compared to the baseline climate, the relative contribution of drought tolerance traits to grain yield will be somewhat variable under climate change, depending on degree of rainfall change and soil water retention characteristics of locations. However, the relative contribution of heat tolerance to grain yield increased with climate change especially at the warmer locations. To enhance and sustain yields of pearl millet under climate change a different combinations of plant traits will be needed at different target locations. The modified CSM-CERES-Pearl Millet model and the virtual crop modeling approach were useful in evaluating such plant genetic traits.

## Figures and Tables

**Table:** 

	Cultivars		
GCs	ICMH 356	Sharda	CIVT
P1	120	120	365
P20	12.7	12	12
P2R	86	15	260
P5	340	360	285
G1	0.7	0.8	0.6
G4	1	1	0.73
PHINT	43	43	43
GT	1.2	1.2	1.2
G5	11	11	11

P2O	Critical photoperiod or the longest day length (in hours) at which development occurs at a maximum rate. At values greater than P2O, the rate of development is reduced
P2R	Extent to which phasic development leading to panicle initiation (expressed in degree days) is delayed for each hour increase in photoperiod above P2O
P5	Thermal time (degree days above a base temperature of 10 °C from beginning of grain filling (3–4 days after flowering) to physiological maturity.
G1	Scaler for relative leaf size
G4	Scaler for partitioning of assimilates to the panicle (head including grain).
PHINT	Phylochron interval; the interval in thermal time (degree days) between successive leaf tip appearances
GT	Tillering coefficient, scalar for relative leaf size, equivalent to G1, but on tillers
G5	Potential grain size, mg

P1 Thermal time from seedling emergence to the end of the juvenile phase (expressed in degree days above a base temperature of 10 °C) during which the plant is not responsive to changes in photoperiod

**Table:** 

Values	Temperature thresholds (°C)			
	Tbase	Topt1	Topt2	Tmax
	PRFTC			
Original values	10	20	40	50
Adjusted values (susceptible and tolerant cultivars)	11	22	35	48
Relative response (unit less)	0	1	1	0
	RGFIL			
Original values	7	22	48	50
Adjusted values (susceptible cultivar)	7	22	27*	60
Adjusted values (tolerant cultivar)	7	22	29	62
Relative response (unit less)	0	1	1	0
	RGSET			
Original values	–	–	–	–
Adjusted values (susceptible cultivar)	− 10	12	33**	39**
Adjusted values (tolerant cultivar)	− 10	12	35	41
Relative response (unit less)	0	1	1	0
	RGLAI			
Original values	8	14	36	46
Adjusted values (susceptible and tolerant cultivars)	8	23	32	42
Relative response (unit less)	0	1	1	0

Tbase: Base temperature; Topt1: Lower optimum temperature; Topt2: Upper optimum temperature; Tmax: Damaging “failure” temperature. *Threshold temperature based on Prasad et al. (2006): ** Threshold temperature based on Gupta et al. (2015).
